# Intelligent algorithms and complex system for a smart parking for vaccine delivery center of COVID-19

**DOI:** 10.1007/s40747-021-00524-5

**Published:** 2021-10-06

**Authors:** Mahdi Jemmali

**Affiliations:** 1 Department of Computer Science and Information, College of Science at Zulfi, Majmaah University, AL-Majmaah, 11952 Saudi Arabia; 2 MARS Laboratory, University of Sousse, Sousse, Tunisia; 3 Department of Computer Science, Higher Institute of Computer Science and Mathematics of Monastir, University of Monastir, 5000 Monastir, Tunisia

**Keywords:** COVID-19, Smart city, Intelligent algorithms, Complex system, Big data, Operations management, 68M20, 90B35

## Abstract

Achieving community immunity against the coronavirus disease 2019 (COVID-19) depends on vaccinating the largest number of people within a specific period while taking all precautionary measures. To address this problem, this paper presents a smart parking system that will help the health crisis management committee to vaccinate the largest number of people with the minimum period of time while ensuring that all precautionary measures are followed, through a set of algorithms. These algorithms seek to ensure a uniform distribution of persons in parking. This paper proposes a novel complex system for smart parking and nine algorithms to address the NP-hard problem. The experimental results demonstrate the performance of the proposed algorithms in terms of gap and time. Applying these algorithms to smart cities to ensure precautionary measures against COVID-19 can help fight against this pandemic.

## Introduction

In December 2019, a respiratory disease was reported in Wuhan, China, which was later designated as severe acute respiratory syndrome coronavirus-2 (SARS-CoV-2, 2019-nCoV) [26]. On February 11, 2020, the World Health Organization (WHO) [20] named it coronavirus disease 2019 (COVID-19). Despite extensive efforts being made globally, COVID-19 has spread rapidly from Wuhan to other areas, infecting an increasing number of people worldwide. Its high contagion rate and the global spread infectivity forced WHO to announce the outbreak the global COVID-19 pandemic on March 11, 2020 [15].

Efforts such as quarantining the infected individuals, physical distancing, lockdowns, closure of schools, and travel restrictions could not contain the outbreak, thus pushing the communities to search for various treatment options [15].

The increasing number of COVID-19 cases has led to critical challenges in people’s lives, as well as overwhelming hospitals, thus threatening global health and medical communities [29]. The outbreak has caused a decline in the economy of most sectors, with massive reductions in certain supply-and-demand aspects of the economy [18]. Despite the various treatment protocols suggested for COVID-19, till the time of writing this paper, there is no effective remedy. Fortunately, many pharmaceutical companies have announced preliminary efficacy results for COVID-19 vaccines. With the availability of the vaccine, many researchers and healthcare organizations believe that distributing the vaccine to those in need will stop the pandemic and suppress its infections [23].

To achieve this goal, vaccines must be distributed on a large scale, which emphasizes the need for policies that include collaborations among government, health organizations, health workers, and public, as well as special resources for vaccine storage and distribution [20, 23, 25]. Once vaccinations start, many issues should be considered, such as continuous vaccine supplies, vaccination strategies, vaccination locations, and commitment to precautionary protocols. Therefore, the most important question at this stage is how to choose the most appropriate place that will meet all these requirements and permit large-scale vaccination simultaneously. To address this question, this research comes as a modest contribution from the scientific research team to join forces with global efforts to provide support to health organizations, to combat the pandemic, and stop its outbreak.

So far, extensive suggestions, recommendations, and challenges regarding large-scale COVID-19 vaccination have been proposed. For example, the authors in [3] explained the importance of collaboration between, and the employment of all available resources of, biotechnology and pharmaceutical agencies to produce the vaccines required to achieve herd immunity. Similarly, the authors in [14] reviewed the challenges faced in developing strategies faced in distributing vaccines among individuals who need them the most. The authors in [16] studied the efficacy of large-scale vaccinations on the community immunity level when life returns to normal. Similarly, the researchers in [28] derived the required interconnected strategies to ensure continuous delivery to achieve large-scale vaccination.

Healthcare organizations require intelligent decision-making technologies, such as machine learning, predictive analytic of big data, and complex systems’ intelligent management dashboards, to provide appropriate real-time instructions to decision-makers, to avoid any errors in the process of large-scale vaccination [4, 27]. For example, the researchers in [7] explained how policy-makers can use artificial intelligence tools to develop healthcare strategies that can be used to combat epidemics in smart cities. In the same context, the researchers in [19] presented an intelligent mechanism that can enhance the level of services provided to the residents of smart cities.

Large-scale vaccination requires the distribution of thousands or millions of vaccine doses to people within a limited period. Providing the required number of vaccine doses requires the establishment of suitable vaccination centers by accounting for the conditions for vaccine storage and the ease of reaching these centers. To address this problem, this study uses the equity distribution method to produce a smart parking system, which can be employed by health authorities to provide vaccine doses to the people who need them the most, without breaking the precautionary standards recommended by the health authorities.

Although many cities around the world are using the traditional drive-through approaches, but these approaches are impractical when large-scale vaccination is required, because the vaccination process may require the provision of a set of predetermined conditions related to the vaccination process itself, such as medical assistance, vaccine storage requirements, and vaccine type selection especially when vaccinations require more than one dose and at different time intervals. Also, some types of vaccines require waiting for some time before the vaccination process ends, these circumstances will cause the accumulation of cars in long queues, and consequently traffic jams and congestions that will disrupt the movement of people and negatively affect the vaccination process.

The studied problem can be very useful on the day of a health event, such as a conference or vaccination during an epidemic. On that day, many people visit the hospital. Indeed, there will be a high demand of vehicle parking and a major risk of its unequal distribution to the people. This problem can be addressed through an appropriate and automatic assignment of vehicles, with the objective of guaranteeing an equal distribution of the number of persons in each parking space. The rest of this paper is organized as follows: the section “Literature review” presents the previous work related to the studied problem, the section “Problem description” provides notations and details that explain the problem, and the section “Parking and vaccination process” presents the novel process of the smart parking and the global algorithm of the process. The section “Proposed algorithms” presents nine new algorithms and the section “Experimental results” discusses the obtained results. Finally, the last section presents the conclusions

## Literature review

Equity distribution methods have been used in several research domains so far. For example, the authors in [1] applied equity algorithms to derive learning strategies that can form the basis for an enhanced education system. In the same context, the authors in [21] used equity algorithms to develop an equity model that statistically demonstrates that equitable access to public services, including education, contributes to building progressive societies that are healthier, richer, and more sophisticated. The authors in [6] demonstrated a framework that integrates machine-learning algorithms to construct housing rent prediction models that monitor housing rental prices, to derive equitable housing policies. In addition, the authors in [17] used equitable distribution methods as a decision support system to control the inflow water-distribution throttle system to achieve an equal distribution of pumped water to city residents. The authors in [13] used integer programming models to implement two frameworks—branch-and-cut and branch-and-price—to address the equitable traveling salesman problem, both of which achieved suitable results for small and medium instances; however, branch-and-bound performed better for large distances. Meanwhile, the authors in [24] proposed a multiobjective evolutionary approach for addressing both simple and complex equitable multiobjective optimization problems. In addition, equity algorithms and large-scale vaccine distribution strategies have been extensively discussed. The authors in [5] used equity constraints as a decision support tool that can be employed by health authorities to ensure equity and effectiveness in balancing the vaccine distribution policy. Similarly, the authors in [22] used mixed-integer linear programming to present an inventory-location optimization model for a uniform influenza vaccine distribution between eight groups of populations based on each group’s coverage rate. An application of the project distribution was proposed in [2, 8, 9], where new algorithms were developed to propose several approximate solutions. However, the authors in [2] developed an exact solution. In addition, an application of network searching for equity distribution was proposed in [10], where several algorithms based essentially on the subset-sum problem, multi-fit method, and dispatching rule method were proposed. Furthermore, several studies (e.g., [11, 12]) have proposed the application of equity distribution to turbine aircraft engines.

## Problem description

The problem considered in this study is described as a large-scale distribution of COVID-19 vaccines within a time schedule of 6 months and without violating the health precautions. This paper presents a vaccine distribution center in the form of a smart parking system, which can serve people, while they are inside their vehicles; these vehicles are fairly distributed between the vaccinating stations within the vaccine center. The main objective is to employ equity distribution algorithms to ensure load balancing between vaccine stations to vaccinate the largest number of people without violating any health protocol and finish the vaccination process during the time limit specified by health organizations. The proposed smart parking center should be built at a location that can be easily accessed, which means that the arrival, vaccination, and departure processes should be all clear and straightforward. Smart parking has numerous portals. A set of these portals is denoted as $\text {Po}$, the portal count is denoted as $n_{{\mathrm{po}}}$, and the index of each portal is denoted as *l*. Therefore, the portal number *l* will be denoted as $\text {Po}_l$. The parking set is denoted as $\text {Pa}$, and the total number of parking space is denoted as $n_{{\mathrm{pa}}}$. The index of each parking space is denoted as *i*, which means that the parking number *i* is denoted as $\text {Pa}_i$.

**Fig. 1:**
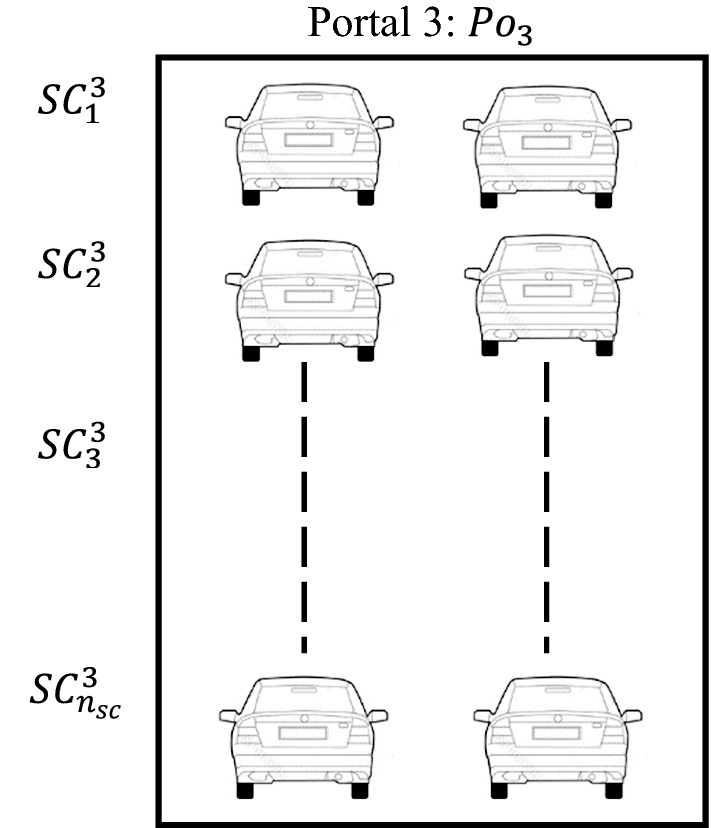
Cameras and sensors’ distribution on gates of portal 3

Each portal has two gates, each of which contains a set of cameras and sensors, denoted by $\text {SC}$, and the total number of cameras and sensors for each gate in a certain portal is denoted as $n_{sc}$. These cameras and sensors aim to collect real-time data on vehicles that enter through each portal’s gates. The collected data are sent to the system control to specify the number of people inside each vehicle. The distribution of cameras and sensors installed on each gate is shown in Fig. 1, which shows their distribution in the gates of portal three.

The cameras and sensors illustrated in Fig. 1 detect the number of persons in each vehicle that passes through the gates of portal 3 at time *t*. Thus, at time 0, these equipment detect the first data reading time $r=1$ of the current vehicles, and then (after all detected vehicles have been scheduled), they detect the next data reading time $r=2$ related to the set of vehicles. This process continues until no vehicles are left to be scheduled. When the data reading on a portal $\text {Po}_l$ are finished, the sensors send a finish declaration to set the variable $\text {finl}$ to $-1.$ The set of vehicles in portal *l* at data reading time *r* is denoted as $\text {Ve}^l (r)$. The vehicle number *m* in portal *l* at data reading time *r* is denoted as $\text {Ve}_m^l(r)$. The set of vehicles at data reading time *r* is denoted as $\text {Ve}(r)$. Thus, $\text {Ve}(r)=\cup _{l=1}^{n_{{\mathrm{po}}}} \text {Ve}^l(r)$.

The number of vehicles at data reading time *r* in portal *l* is denoted as $n_{{\mathrm{ve}}}^l(r)$, whereas the number of vehicles at the data reading time *r* is denoted as $n_{{\mathrm{ve}}}(r)$. Consequently, $n_{{\mathrm{ve}}}(r)=\sum _{l=1}^{n_{{\mathrm{po}}}}n_{{\mathrm{ve}}}^l(r)$. Each element of the set $\text {Ve}(r)$ is denoted as $\text {Ve}_j(r)$ with $j=\{1,\ldots ,n_{{\mathrm{ve}}}(r)\}$.

By correspondence of the vehicles, we denoted the number of persons as follows.

The number of persons in portal *l* at data reading time *r* is denoted as $\text {Pe}^l (r)$. The number of persons in the vehicle number *m* in portal *l* at data reading time *r* is denoted as $\text {Pe}_m^l(r)$. The number of persons at data reading time *r* is denoted as $\text {Pe}(r)$.

The total number of persons scheduled for parking $\text {Pa}_i$ is known as the load of each parking, which is denoted as $L_i(r)$. When a vehicle $\text {Ve}_m^l(r)$ is scheduled for parking $\text {Pa}_i$, the cumulative load is denoted by $L_i^{l,m}(r)$. The gates are denoted as $G_k^l$, where *l* is the number of portals and *k* is the gate number for that portal. Each portal has $n_g$ gates. These gates will be used to organize vehicles and prevent overcrowding.

**Fig. 2:**
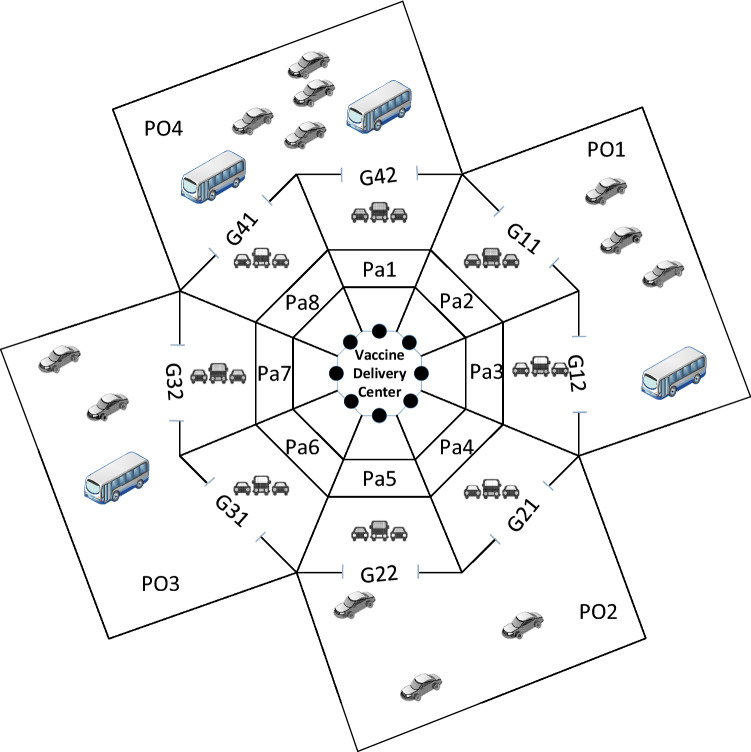
The design of the smart parking for the COVID-19 vaccine distribution center

This section presents a numerical example to demonstrate the basic concept of the smart parking center. Suppose that there are four portals and eight parking spaces. Portal $\text {Po}_1$ contains four vehicles, $\text {Po}_2$ contains three vehicles, $\text {Po}_3$ contains three vehicles, and $\text {Po}_4$ contains six vehicles. Thus, in total, 16 vehicles need to be scheduled for eight parking spaces. Let us assume that $r=5$ and that each portal has two gates (see Fig. 2).

The distribution of given vehicles on the portals is detailed as follows:

- In $$\text {Po}_1$$ there is the set of vehicles $$\text {Ve}^1(5)=\{\text {Ve}_1^1(5),$$$$\text {Ve}_2^1(5), \text {Ve}_3^1(5),\text {Ve}_4^1(5),\text {Ve}_5^1(5)\}$$.
- In $$\text {Po}_2$$ there is the set of vehicles $$\text {Ve}^2(5)=\{\text {Ve}_1^2(5),\text {Ve}_2^2(5),$$$$\text {Ve}_3^2(5),\text {Ve}_4^2(5)\}$$.
- In $$\text {Po}_3$$ there is the set of vehicles $$\text {Ve}^3(5)=\{\text {Ve}_1^3(5),\text {Ve}_2^3(5),$$$$\text {Ve}_3^3(5)\}$$.
- In $$\text {Po}_4$$ there is the set of vehicles $$\text {Ve}^4(5)=\{\text {Ve}_1^4(5),\text {Ve}_2^4(5),$$$$\text {Ve}_3^4(5),\text {Ve}_4^4(5),\text {Ve}_5^4(5),\text {Ve}_6^4(5)\}$$.

The number of persons in each vehicle at each portal is given in Table 1.

**Fig. 3:**
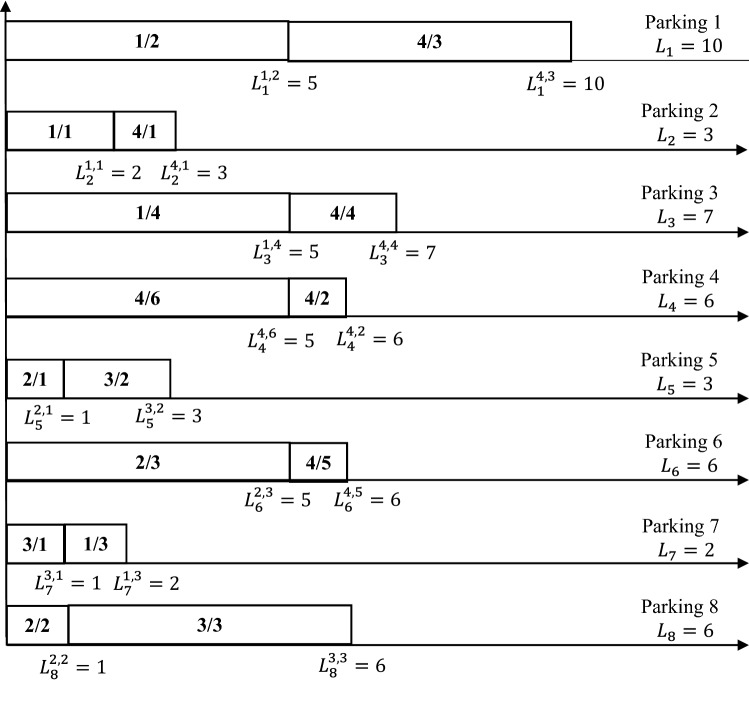
Schedule of vehicles *l*, *m* on parking

The problem is to search for an appropriate schedule to distribute the set of vehicles ${\text {Ve}^1(5),\text {Ve}^2(5),\text {Ve}^3(5),\text {Ve}^4(5)}$ in the eight parking spaces, ensuring fair distribution. Figure 3 illustrates the schedule of the vehicles to be parked. Seeking simplicity, $\text {Ve}_m^l(5)$ will be replaced by *l*/*m*. For example, in parking 1, there are 1/2 and 4/3. This is meaning the vehicle in portal 1 number 2 (denoted by 1/2) and vehicle in portal 4 number 3 (denoted by 4/3) are parked in parking 1. $L_1^{1,2}$ is the load of parking after vehicle 1/2 is parked.

As shown in Fig. 3, all parking spaces contain the same number of vehicles. However, all parking spaces do not have the same number of persons. For example, parking $\text {Pa}_1$ has ten persons, parking spaces $\text {Pa}_2$ and $\text {Pa}_5$ have three persons each, and $\text {Pa}_7$ has only two persons each. The main goal here is to seek an algorithm that ensures a fair distribution (equitable) of persons for each parking. To achieve this goal, we must minimize the parking-space load variations by minimizing the difference between each parking load and the minimum load. For certain reading data *r*, the gap value in the number of persons for each parking space is calculated using Eq. 1

1 $$\begin{aligned} g(r)=\sum _{i=1}^{i=n_{{\mathrm{pa}}}}[L_i(r)-L_{{\mathrm{min}}}(r)]. \end{aligned}$$

The main objective of this study is to minimize *g*(*r*) to ensure an equitable distribution of persons for each parking space, which indicates an equitable group of people for each vaccination center; this will be the primary achievement of this study.

### Proposition 1

The objective function of the studied problem can be rewritten as follows: $g(r)=\sum _{i=1}^{i=n_{{\mathrm{pa}}}}L_i(r)-n_{{\mathrm{pa}}}\times L_{{\mathrm{min}}}(r)$.

### Proof

Based on Eq. 1$g(r)=\sum _{i=1}^{i=n_{{\mathrm{pa}}}}[L_i(r)-L_{{\mathrm{min}}}(r)]$. Thus, $g(r)=\sum _{i=1}^{i=n_{{\mathrm{pa}}}}L_i(r)-\sum _{i=1}^{i=n_{{\mathrm{pa}}}}L_{{\mathrm{min}}}(r)$. On the other hand, $\sum _{i=1}^{i=n_{{\mathrm{pa}}}}L_{{\mathrm{min}}}(r)=n_{{\mathrm{pa}}}\times L_{{\mathrm{min}}}(r)$. Finally, we have $g(r)=\sum _{i=1}^{i=n_{{\mathrm{pa}}}}L_i(r)-n_{{\mathrm{pa}}}\times L_{{\mathrm{min}}}(r)$. $\square$

When we apply the calculations of *g*(*r*) to the schedule given in Fig. 3, the first step is to determine $L_{{\mathrm{min}}}(r)$. Figure 3 shows that $L_{{\mathrm{min}}}(r)=2$. Thus, $g(r)=[(10+3+7+6+3+6+2+6)-(8\times 2)]=27$. The objective is to reach a gap value of less than 27. For example, consider moving vehicle $\text {Ve}_3^4$ from parking $\text {Pa}_1$ to parking $\text {Pa}_7$. Consequently, we will have a new value of $L_{{\mathrm{min}}}(r)=3$ and the new gap value will be $g(r)=[(5+3+7+6+3+6+7+6)-(8\times 3)]=19$. Which means that we have won 8 units compared to the old schedule presented in Fig. 3 because of moving $\text {Ve}_3^4$ from $\text {Pa}_1$ to parking $\text {Pa}_7$.

## Parking and vaccination process

**Fig. 4:**
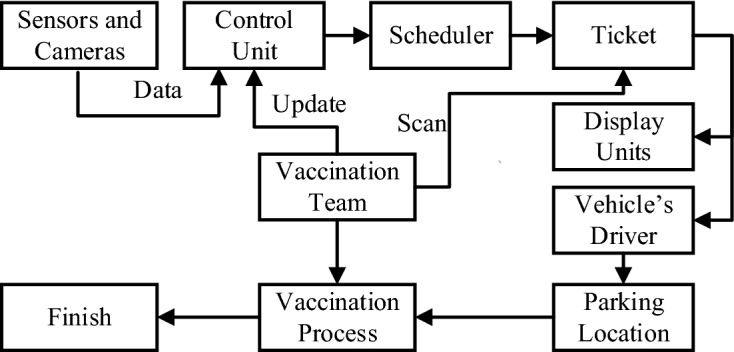
Parking and vaccination process diagram

As described above, each portal is equipped with cameras and sensors that send the captured data about each vehicle to the smart parking control unit. This unit uses these data to derive information regarding the number of persons in each vehicle in each portal, and then sends this information to the scheduler. The scheduler applies the proposed algorithms to the received data to generate a new schedule, which is sent to the control unit. Based on the results of the new schedule, the control unit issues an order to transfer vehicles to new parking locations. This order contains the required directions to guide the driver of the vehicle to the specified parking location. This order is in the form of a display ticket, which is submitted to the vehicle’s driver and has the required directions to guide the driver to the specified parking location. Displays are mounted on the available parking locations, which indicate the vehicle’s plate number and the assigned location for each vehicle. At each specified location, a team of healthcare specialists delivers the COVID-19 vaccine to the people inside each vehicle. Then, the vaccine team scans the vehicle’s previously received ticket to update the control unit data, which updates the scheduler. This scheduler generates a new schedule, as shown in Fig. 4. After receiving the vaccine, the vehicles leave the vaccination center through the specified exit gates.

**Table 1:** The number of persons $\text {Pe}_m^l(5)$ in each portal *l* and each vehicle $\text {Ve}_m^l(5)$

*l*/*m*	1	2	3	4	5	6
1	2	5	2	5	–	–
2	1	1	5	–	–	–
3	1	2	5	–	–	–
4	1	1	5	2	1	5

The components presented in Fig. 4 are explained as follows.

- Sensors and cameras: The cameras and sensors aim to collect real-time data about the vehicles that enter through each portal’s gates. The collected data contain vehicle entrance time, vehicle plate number, vehicle size, and the number of people inside each vehicle.
- Control unit: The control unit processes the received data to specify the number of people inside each vehicle and identify the vehicle size at each gate based on the sizes defined by the smart parking system, which are $T_1$, $T_2$, and $T_3$ (see “Tested instances”). It performs the required calculations to provide the scheduler with the required information to generate the required schedules.
- Scheduler: This part of the system generates a schedule that uses a set of complex algorithms that are developed to solve the problem of distributing vehicles to different parking locations while ensuring the equity distribution of people inside each vaccine center. The generated schedule defines the destination of each vehicle by specifying the parking number to which each vehicle must drive.
- Ticket: Based on the scheduler output, this part generates a dataset that contains the vehicle plate number, time of entry, number of people inside each vehicle, parking location number, and vaccine center to which the vehicle must drive. The generated data are in the form of a printed ticket delivered to the vehicle driver and in an electronic file that is displayed on screens designated for this purpose and distributed within the center in several carefully chosen locations.
- Display units: Display units are distributed on all paths leading to different parking locations. Each parking location is assigned an asset of designated display units that display information regarding this parking location. The display units display the number of parking locations and vehicles assigned to that parking location. Moreover, they can be used to indicate restrictions and guidelines regarding the vaccination process.
- Vehicle driver: The role of the vehicle driver is to follow the displayed instructions and to use the given ticket to reach the parking location assigned to his vehicle.
- Parking location: The place represents the destination of each vehicle where a vaccine delivery center is developed to provide the vaccine to those who deserve it. Each parking location is identified by a unique number, which is displayed to make it easy for users to locate it.
- Vaccination team: Receives ticket data for each vehicle, which contains the vehicle plate, the number of people in each vehicle, and the parking location. The vaccination team must prepare the required instruments for the vaccination process, such as the required number of vaccine doses, syringes, and medical adhesives.
- Vaccination process: Once the vehicle reaches the specified parking location, the vaccination process starts by registering the required data of the people who will be vaccinated and scanning the vehicle that is given a ticket after the vaccination process is completed, to update the scheduler data. The updated data are sent to the control unit to update the scheduler and to the analyzer to perform the required data analysis.
- Analyzer: The analyzer analyzes the received data to obtain information about the vaccination process, such as the total number of processed requests, the status of all smart parking components, the number of vehicles passing through each gate, the number of vehicles at each parking location, and the number of serviced vehicles at each parking location. In addition, the information about the vaccinated people, such as the total number of vaccinated people, age, gender, and any other useful information regarding the vaccination process, is sent to the dashboard of the authorities that carry out the vaccination process.
- Vaccination agency dashboard: This dashboard aims to control and monitor the smart parking vaccine center to make the required intervention when necessary and to generate the required reports regarding the status of the smart parking vaccine center and the vaccination process.

The $\text {SC}$ sends information frequently to the control unit, which is responsible for storing data including the number of portals, and an array contains the $\text {Pe}(r)$ values in the buffer. The control unit receives data from the $\text {SC}$ and determines the portal that contains the ready information.

The passage of cars in the lanes does not guarantee that the first lane will be ready before the second lane; the second passage might be ready before the first passage. Therefore, the information must be filtered by a control unit.

Let *Read*() be a function that is responsible for reading data from the buffer and *Schedule*() be the function responsible for executing appropriate algorithms to derive the scheduling of the data stored in the buffer. $Send_d()$ is the function that is responsible for sending to the display devices the appropriate parking information required for each waiting vehicle to reach the right parking location. Let $Send_{tk}()$ be the function responsible for issuing the ticket to the waiting vehicle. This ticket holds the identification of the vehicle, the number of passengers in the vehicle, and the exact parking location. Based on the above functions, the algorithm responsible for organizing vehicles in the proposed smart parking is presented as Algorithm 1. 
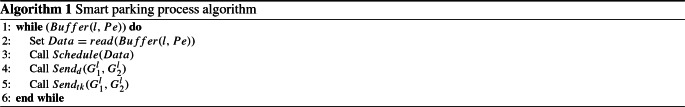


## Proposed algorithms

This section presents the algorithms developed to address the studied problem. These algorithms are based on several techniques. The first technique is the dispatching rules. Algorithms’ development is performed by employing randomization and iterative methods. In addition, the clustering method, which is based on the division of the set of vehicles into groups and applying the randomization method to choose between these groups to schedule vehicles in parking, is used. In total, nine algorithms are developed and implemented.

### Decreasing order-based algorithm (DA)

For this algorithm, the first step is to order all vehicles according to the decreasing order of the number of people present in the vehicle. Then, we assign the first vehicle to the parking space with the minimum number of people, and so on.

### Iterative random parking choice algorithm (IR)

This algorithm is based on the iterative and random (IR) method. For each vehicle, the parking space is chosen randomly and the algorithm is looped *lm* times. For each time, the random function yields a new result and the best solution is selected. The manner in which we randomly select the parking space is based on three methods. The first method randomly chooses the parking space without any constraint. In the second method, for each vehicle *j*, parking is randomly selected from the list of available parking spaces, excluding the space that receives the last vehicle $j-1$. In the third method, for each vehicle, the parking space is randomly selected from the list of available spaces, excluding the most loaded parking. In addition, three variants are adopted in this algorithm. These variants are based on the initial order of the vehicles. The first variant is the choice of the vehicle according to the vehicle index. The second variant is to initially order the vehicles according to the increasing order of the number of persons in the vehicle. The third variant is to initially order the vehicles according to the decreasing order of the number of persons in the vehicle. The best solution is selected after the execution of all variants.

Finally, for each method described above and for each variant, the procedure is looped several times. The procedures of the first, second, and third methods are denoted as M1(), M2(), and M3(), respectively. The corresponding returned parking gaps *g*(*r*) for each method are represented as $g_1$, $g_2$, and $g_3$, respectively. Hereafter, we denote as In() the procedure that sorts vehicles according to the increasing order of their number of persons. De() is the procedure that sorts vehicles according to the decreasing order of their number of persons. In practice, *lm* is fixed to 2000.

The instructions of algorithm IR are described in Algorithm 2. 
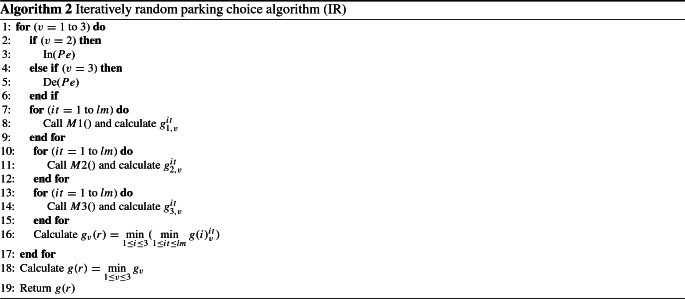


### Iterative random algorithm on the least-loaded parking (IL)

This algorithm is based on the IR method. For each vehicle, the parking space is chosen randomly. The algorithm is looped by *lm* times. For each time, the random function yields a new result and the best solution is selected. The manner in which we randomly choose parking is based on the least-loaded parking. For each vehicle, we randomly choose a parking space among the two least-loaded parking spaces. The three variants described in the section “Iterative random parking choice algorithm (IR)” are also applied to this algorithm. For each variant, the random procedure is looped several times. The choice of the iteration number is fixed at *lm* (see the section “Iterative random parking choice algorithm (IR)”). We denote the procedure responsible for finding the two least-loaded parking spaces as Call least-loaded2(). These parking spaces are stored in variables $L_1$ and $L_2$. In addition, we denote the function that randomly returns a parking space among two functions given as input by Rand(). The Sched() procedure is responsible for scheduling the vehicle on the selected parking $L_r$. In practice, *lm* is fixed to 2000

The instructions of algorithm IL are described in Algorithm 3. 
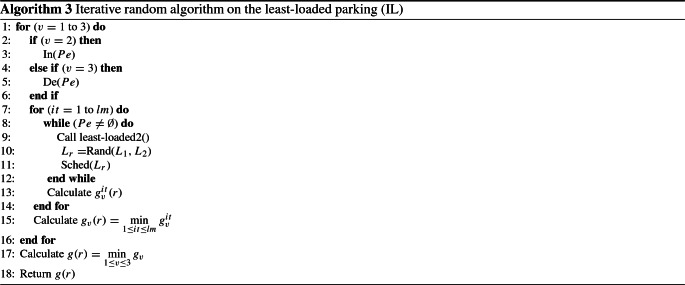


### M-vehicles applying randomized DA and the rest applying the DA algorithm (MR)

This algorithm is divided into two steps. The first step is to schedule the first part of the vehicles according to randomized DA and the second part according to DA. The manner in which we choose the parts is as follows. We denote by $\text {MU}$ an integer that presents the multiplier that fixes the first part of the vehicles to be scheduled according to randomized DA. In fact, the first $\text {MU}\times n_{{\mathrm{pa}}}$ vehicles are scheduled according to randomized DA and the remaining vehicles are scheduled according to DA. This algorithm is denoted as RD. For the randomized DA algorithm, the randomization approach is based on choosing a probability $\beta$ to select the vehicle that has the largest number of people and $1-\beta$ for the next vehicle. The instructions of randomized DA, which is denoted as RMDA(.), is illustrated in Algorithm 4, where *M* (the input of the function) is the part of the vehicles that is determined by the multiplier $\text {MU}$. Hereafter, the procedure Sch(*j*) is responsible for scheduling vehicle *j* on the parking that has the minimum number of persons. 
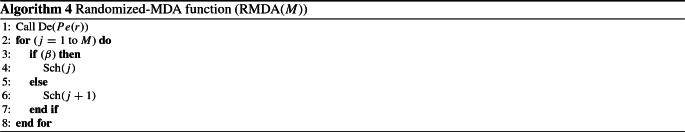


$\text {lmt}$ is the limit number that cannot be exceeded for the iteration of the multiplier. In practice, we select $\text {lmt}=50$. RestSch() is a function that schedules the remaining vehicles according to the DA algorithm. For each *M* value, a looping of *lm* times is done to calculate the gap related to *M* and the iteration counter *it* denoted by $g_{M}^{it}$. In practice, *lm* is fixed to 2000.

The steps of instructions of Algorithm MR is illustrated in Algorithm 5. 
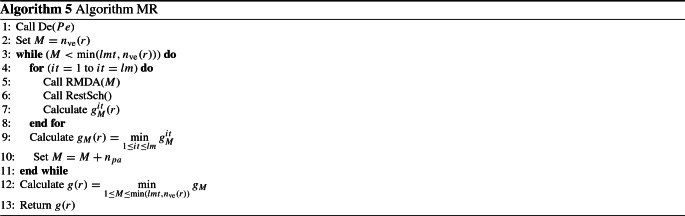


### Clustering algorithm based on two sets (C2S)

The clustering method is used in this algorithm by choosing two sets of vehicles that can create the classification. The scheduling is applied based on the chosen vehicle among those in the determined sets. These sets are denoted by $S_1$ and $S_2$. The vehicles are sorted according to the non-increasing order of their number of persons. The $S_1$ and $S_2$ sets are constructed as follows. Initially, $S_1$ and $S_2$ are empty. The first vehicle is selected and assigned to the first set $S_1$. Subsequently, the second vehicle is selected and assigned to the set that contains the minimum cumulative number of persons, and so on. Now, the sets $S_1$ and $S_2$ are well defined. Randomization is applied between the two sets to choose a vehicle to be scheduled on the parking that has the minimum number of persons. The randomization performed to select one of the sets is based on the generation of a probability $\alpha$ to pick the first vehicle from $S_1$ and probability $1-\alpha$ to pick the first vehicle from $S_2$. This procedure is repeated *lm* times. The instructions of the developed heuristic are detailed in Algorithm 6. 
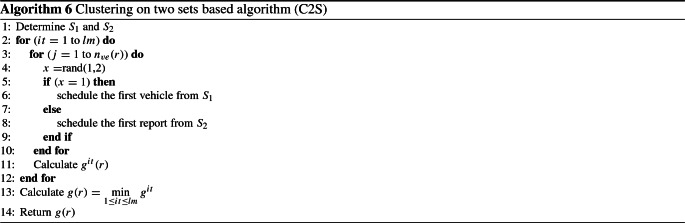


### Clustering based on three sets (C3S)

This algorithm is based on the same concept as that described in the section “Clustering algorithm based on two sets (C2S)”. While the C2S algorithm chooses two sets to be defined, in the C3S algorithm, three sets are chosen. Thus, after defining the latter sets by applying the randomization method, the selection to schedule the vehicles will be made among these three sets.

### Randomized decreasing order-based algorithm (RD)

In this algorithm, we apply randomization to the first three vehicles that have the maximum number of persons. In the first step, the vehicles are ordered according to the non-increasing order of their number of persons. Subsequently, we apply the probability of choosing among the first three vehicles. The first vehicle is chosen with probability $\theta$ and the second with probability $\gamma$, where $\gamma <\theta$ and $\gamma +\theta <1$. The third vehicle is chosen with probability $1-\gamma -\theta$. This procedure is repeated *lm* times and the best solution is chosen.

### Part of vehicles applying RMDA and the remaining applying DA algorithm ($\hbox {RD}_\alpha$)

This algorithm is based on the concept that the entire set of vehicles is divided into two groups. The first group $G_1$ is scheduled according to the RMDA function (see Algorithm 4). In the latter algorithm, we fix $M=n_{{\mathrm{ve}}}(r)\times \alpha$. The second group of vehicles, $G_2$, is scheduled according to DA. In practice, the probability $\alpha$ is in the range of $\{0.1,0.2,0.3,0.4,0.5,0.6,0.7,0.8,0.9\}$. For each value of $\alpha$, we loop the algorithm *lm* times and select the best solution. The probability $\beta$ is the criterion of the choice of the vehicle among the first two vehicles that contain the maximum number of persons.

The instructions of the developed heuristic are detailed in Algorithm 7. 
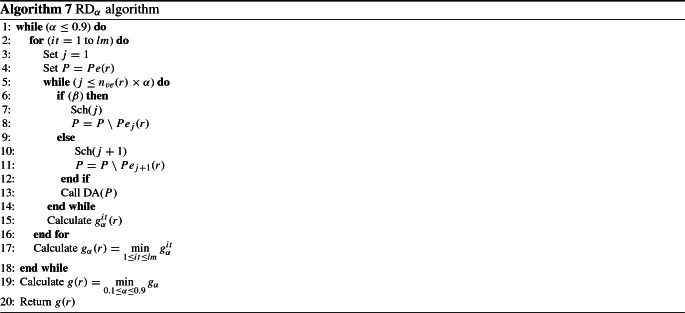


### Part of vehicles applying RMDA and the remaining applying random algorithm ($\hbox {RR}_\alpha$)

This algorithm is based on the same concept as that of algorithm $\hbox {RD}_\alpha$. The difference lies in the scheduling of the second group, which is scheduled to apply a randomized algorithm. This means that the vehicle from group $G_2$ is chosen randomly.

## Experimental results

To evaluate the performance of the proposed algorithms, an extensive experimental study is conducted, where all developed algorithms are coded in C++ and run on Intel(R) Core(TM) i7-3770U CPU @ 3.40 GHz 3.40 GHz and 8 GB RAM. The proposed algorithms are tested and run on a set of test problems, which are detailed below. In the next subsections, we describe the instance generation and the results.

### Tested instances

In this subsection, the generation of instance tests is described. First, the various vehicles chosen to be set in this study are presented. In this study, the following three types of vehicles are selected:

- Type 1 ($T_1$): mini, grand car: max capacity is 9
- Type 2 ($T_2$): Minibus: max capacity is 19,30
- Type 3 ($T_3$): Bus: max capacity is 40,50,70.

In this paper, we choose to apply algorithms on eight principle categories ($Ct_z$ with $z\in \{1,\ldots ,8\}$). The percentage of each type of vehicle distribution for each category is shown in Table 2.

**Table 2:** Percentage of each type of vehicle distribution for each category

	$T_1$ (%)	$T_2$ (%)	$T_3$ (%)
$Ct_1$	100	0	0
$Ct_2$	0	100	0
$Ct_3$	0	0	100
$Ct_4$	70	10	20
$Ct_5$	5	70	25
$Ct_6$	5	25	70
$Ct_7$	0	60	40
$Ct_8$	0	40	60

For each category, we determine the number of persons present in each vehicle, $\text {Pe}_j(r)$, by applying a uniform distribution *U*[.]. Types 2 and 3 have two and three diversities, respectively. The number of possibilities for generating $\text {Pe}_j^l(r)$ is 6. Each possibility is denoted as a class. Table 3 illustrates the distribution of classes for each type of $\text {Pe}_j(r)$ generation.

**Table 3:** Distribution of classes for each kind of generation of $\text {Pe}_j(r)$

	$T_1$	$T_2$	$T_3$
$C_1$	*U*[1, 9]	*U*[9, 19]	*U*[10, 40]
$C_2$	*U*[1, 9]	*U*[9, 19]	*U*[20, 50]
$C_3$	*U*[1, 9]	*U*[9, 19]	*U*[30, 70]
$C_4$	*U*[1, 9]	*U*[10, 30]	*U*[10, 40]
$C_5$	*U*[1, 9]	*U*[10, 30]	*U*[20, 50]
$C_6$	*U*[1, 9]	*U*[10, 30]	*U*[30, 70]

The choice of *U*[9, 19] for the second type is fixed, because we suppose that the minibus will has at minimum 9 persons among 19. The same concept is applied to Type 3. Now, for categories $Ct_z$ with $z\in \{4,\ldots ,8\}$, we apply the 6 classes. This is given as $5\times 6=30$ varieties. For $Ct_1$, the percentage for choosing $T_2$ and $T_3$ are 0. This means that there is no utility to iterate *U*[1, 9] six times. Indeed, we have only one variety for this category. For $Ct_2$, we apply only two varieties that are related to the different intervals *U*[9, 19] and *U*[10, 30]. This means that the corresponding classes can only be $C_1$ and $C_4$. For $Ct_3$, we apply only three varieties that are related to the different intervals *U*[10, 40], *U*[20, 50], and *U*[30, 70]. This is means that the corresponding classes can be only $C_1$, $C_2$, and $C_3$. Table 4 illustrates the exceptional distribution category classes described above.

**Table 4:** Exceptional distribution category classes

Category	Classes	Varieties
$Ct_1$	$C_1$	1
$Ct_2$	$C_1,C_4$	2
$Ct_3$	$C_1,C_2,C_3$	3

**Table 5:** Overview of all algorithm results

	DA	IR	IL	MR	C2S	C3S	RD	$\hbox {RD}_{\alpha }$	$\hbox {RR}_\alpha$
$\text {Pge}$	49.0%	32.7%	74.3%	67.8%	90.6%	94.0%	73.7%	73.4%	81.4%
$\text {Ag}$	0.41	0.58	0.19	0.24	0.06	0.04	0.19	0.20	0.12
$\text {Te}$	–	0.138	0.072	0.311	0.022	0.028	0.076	0.353	0.361

**Table 6:** Variation of $\text {Ag}$ and $\text {Te}$ according to $n_{{\mathrm{ve}}}(r)$ for all algorithms

$n_{{\mathrm{ve}}}(r)$	DA		IR		IL		MR		C2S		C3S		RD		$\hbox {RD}_{\alpha }$		$\hbox {RR}_{\alpha }$	
	$\text {Ag}$	$\text {Te}$	$\text {Ag}$	$\text {Te}$	$\text {Ag}$	$\text {Te}$	$\text {Ag}$	$\text {Te}$	$\text {Ag}$	$\text {Te}$	$\text {Ag}$	$\text {Te}$	$\text {Ag}$	$\text {Te}$	$\text {Ag}$	$\text {Te}$	$\text {Ag}$	$\text {Te}$
20	0.48	–	0.50	–	0.18	0.01	0.15	–	0.06	–	0.06	–	0.12	–	0.14	0.01	0.08	0.02
50	0.53	–	0.60	–	0.19	0.02	0.25	0.03	0.05	–	0.07	0.01	0.26	0.01	0.24	0.04	0.17	0.05
100	0.41	–	0.59	–	0.20	0.04	0.31	0.07	0.04	0.01	0.04	0.01	0.24	0.02	0.24	0.09	0.14	0.11
300	0.22	–	0.56	–	0.16	0.11	0.21	0.43	0.05	0.03	0.01	0.04	0.16	0.11	0.16	0.50	0.09	0.52
500	0.27	–	0.54	–	0.17	0.18	0.27	1.01	0.12	0.06	0.01	0.08	0.22	0.24	0.23	1.12	0.07	1.11

**Table 7:** Variation of $\text {Ag}$ and $\text {Te}$ according to $n_{{\mathrm{pa}}}$ for all algorithms

$n_{{\mathrm{pa}}}$	DA		IR		IL		MR		C2S		C3S		RD		$\hbox {RD}_{\alpha }$		$\hbox {RR}_{\alpha }$	
	$\text {Ag}$	$\text {Te}$	$\text {Ag}$	$\text {Te}$	$\text {Ag}$	$\text {Te}$	$\text {Ag}$	$\text {Te}$	$\text {Ag}$	$\text {Te}$	$\text {Ag}$	$\text {Te}$	$\text {Ag}$	$\text {Te}$	$\text {Ag}$	$\text {Te}$	$\text {Ag}$	$\text {Te}$
2	0.12	–	0.00	0.13	0.00	0.03	0.00	0.70	0.00	0.02	0.00	0.02	0.00	0.07	0.00	0.32	0.00	0.32
3	0.50	–	0.01	0.11	0.01	0.04	0.21	0.48	0.02	0.02	0.00	0.02	0.14	0.07	0.17	0.33	0.00	0.33
4	0.30	–	0.26	0.11	0.05	0.06	0.09	0.36	0.00	0.02	0.01	0.03	0.06	0.07	0.07	0.34	0.01	0.34
5	0.27	–	0.59	0.12	0.08	0.06	0.06	0.28	0.02	0.02	0.03	0.03	0.04	0.07	0.03	0.35	0.04	0.36
6	0.48	–	0.72	0.13	0.28	0.07	0.40	0.25	0.11	0.02	0.04	0.03	0.33	0.08	0.35	0.35	0.16	0.36
7	0.53	–	0.81	0.14	0.34	0.08	0.39	0.22	0.13	0.02	0.06	0.03	0.35	0.08	0.34	0.36	0.20	0.37
8	0.51	–	0.85	0.15	0.37	0.09	0.45	0.20	0.13	0.02	0.05	0.03	0.40	0.08	0.42	0.37	0.24	0.38
9	0.48	–	0.88	0.17	0.34	0.10	0.41	0.17	0.13	0.02	0.06	0.03	0.36	0.08	0.37	0.37	0.25	0.39
10	0.23	–	0.89	0.19	0.14	0.11	0.14	0.14	0.04	0.03	0.08	0.03	0.10	0.08	0.07	0.38	0.10	0.39

Thus, in total, we have $30+6=36$ varieties to generate instances, for each of which we generate five instances. Thus, 180 instances need to be generated. The number of vehicles $n_{{\mathrm{ve}}}(r)$ is in $\{20,50,100,300,500\}$. While the number of parking $n_{{\mathrm{pa}}}(r)$ is in $=\{2,3,4,5,6,7,8,9,10\}$. For each $n_{{\mathrm{ve}}}(r)$ and each $n_{{\mathrm{pa}}}(r)$ value, we generate the 180 instances. Finally, in total, we have $180\times 5\times 9=8100$ instances. The performance of each proposed algorithm detailed in the section “Proposed algorithms” is verified using the following indicators:

- $A_*$ The best (minimum) value obtained after running all algorithms.
- *A* The studied algorithm.
- $\text {Pge}$ The percentage of instances when $A_*=A$.
- $$G=\frac{A-A_*}{A}$$, if $$A=0$$ then $$G=0$$.
- $\text {Ag}$ The average of *G* for a determined instances.
- $\text {Te}$ The time required to execute an algorithm for the corresponding instances. This time is measured in seconds and we recorded as “–” if the time is less than 0.001 s.

### Discussion of results

In this subsection, we illustrate all results achieved using the developed algorithms. Table 5 presents an overview of all algorithm results. This table shows that the best algorithm is C3S with a percentage of 94%, an average gap of 0.04, and a running time of 0.028 s. The second-best algorithm is C2S, with a percentage of 90.6%, an average gap of 0.06, and a running time of 0.022 s. The minimum $\text {Pge}$ value of 32.7% is obtained for the IR algorithm with a maximum gap of 0.58. The algorithm that is the most time-consuming is $\hbox {RR}_\alpha$, with an average time of 0.361 s.

**Table 8:** Variation of $\text {Ag}$ and $\text {Te}$ according to *Ct* for all algorithms

*Ct*	DA		IR		IL		MR		C2S		C3S		RD		$\hbox {RD}_{\alpha }$		$\hbox {RR}_{\alpha }$	
	$\text {Ag}$	$\text {Te}$	$\text {Ag}$	$\text {Te}$	$\text {Ag}$	$\text {Te}$	$\text {Ag}$	$\text {Te}$	$\text {Ag}$	$\text {Te}$	$\text {Ag}$	$\text {Te}$	$\text {Ag}$	$\text {Te}$	$\text {Ag}$	$\text {Te}$	$\text {Ag}$	$\text {Te}$
1	0.05	–	0.34	0.14	0.02	0.07	0.02	0.31	0.01	0.02	0.00	0.03	0.02	0.07	0.02	0.35	0.01	0.36
2	0.44	–	0.57	0.14	0.25	0.07	0.35	0.31	0.11	0.02	0.04	0.03	0.33	0.07	0.35	0.35	0.10	0.36
3	0.51	–	0.63	0.14	0.29	0.07	0.38	0.31	0.15	0.02	0.06	0.03	0.36	0.08	0.37	0.35	0.16	0.36
4	0.05	–	0.47	0.14	0.02	0.07	0.02	0.31	0.01	0.02	0.00	0.03	0.02	0.08	0.02	0.35	0.00	0.37
5	0.51	–	0.61	0.14	0.19	0.07	0.21	0.31	0.03	0.02	0.04	0.03	0.15	0.08	0.13	0.35	0.14	0.36
6	0.49	–	0.61	0.14	0.18	0.07	0.26	0.31	0.03	0.02	0.05	0.03	0.15	0.08	0.14	0.35	0.14	0.36
7	0.49	–	0.61	0.14	0.24	0.07	0.31	0.31	0.08	0.02	0.05	0.03	0.26	0.08	0.28	0.36	0.16	0.36
8	0.51	–	0.63	0.14	0.26	0.07	0.35	0.31	0.09	0.02	0.06	0.03	0.30	0.08	0.30	0.35	0.18	0.36

**Table 9:** Variation of $\text {Ag}$ and $\text {Te}$ according to *C* for all algorithms

*C*	DA		IR		IL		MR		C2S		C3S		RD		$\hbox {RD}_{\alpha }$		$\hbox {RR}_{\alpha }$	
	$\text {Ag}$	$\text {Te}$	$\text {Ag}$	$\text {Te}$	$\text {Ag}$	$\text {Te}$	$\text {Ag}$	$\text {Te}$	$\text {Ag}$	$\text {Te}$	$\text {Ag}$	$\text {Te}$	$\text {Ag}$	$\text {Te}$	$\text {Ag}$	$\text {Te}$	$\text {Ag}$	$\text {Te}$
1	0.35	–	0.56	0.19	0.20	0.10	0.25	0.45	0.08	0.03	0.02	0.04	0.22	0.11	0.23	0.53	0.10	0.54
2	0.40	–	0.62	0.19	0.21	0.10	0.27	0.45	0.09	0.03	0.03	0.04	0.22	0.11	0.23	0.53	0.13	0.54
3	0.39	–	0.63	0.19	0.17	0.10	0.25	0.45	0.08	0.03	0.05	0.04	0.16	0.11	0.15	0.53	0.10	0.54
4	0.40	–	0.61	0.19	0.21	0.10	0.30	0.46	0.08	0.03	0.03	0.04	0.24	0.11	0.26	0.53	0.14	0.54
5	0.40	–	0.62	0.19	0.21	0.10	0.29	0.45	0.06	0.03	0.03	0.04	0.22	0.11	0.23	0.53	0.15	0.54
6	0.40	–	0.64	0.19	0.19	0.10	0.26	0.45	0.05	0.03	0.04	0.04	0.19	0.12	0.19	0.53	0.14	0.54

Table 6 presents the variation in $\text {Ag}$ and $\text {Te}$ according to $n_{{\mathrm{ve}}}(r)$ for all algorithms. It shows that the minimum average gap of 0.01 for algorithm C3S is obtained when $n_{{\mathrm{ve}}}(r)=\{300,500\}$. In addition, the maximum Ag value of 0.60 is obtained by the IR algorithm when $n_{{\mathrm{ve}}}(r)=50$. Note that the running time increases when $n_{{\mathrm{ve}}}(r)$ increases for the algorithms. Although the algorithm that is most time-consuming is $\hbox {RR}_\alpha$ (Table 5), the algorithm that takes the maximum running time reaching 1.12 s when $n_{{\mathrm{ve}}}(r)=500$ is $\hbox {RD}_\alpha$.

Table 7 presents the variation in $\text {Ag}$ and $\text {Te}$ according to $n_{{\mathrm{pa}}}$ for all algorithms. For algorithm C3S, the average gap is less than 0.01 when $n_{{\mathrm{pa}}}(r)=\{2,3\}$. The maximum average gap for C3 is 0.08, obtained when npa(r) = 10, and that for C2S is 0.13, obtained when $n_{{\mathrm{pa}}}(r)=\{7,8,9\}$. For the algorithms C2S and C3S, the running time is approximately 0.02 s. However, for $\hbox {RR}_{\alpha }$, the running time reaches 0.39 s when $n_{{\mathrm{pa}}}(r)=\{9,10\}$. The running time increases with $n_{{\mathrm{pa}}}(r)$ for all algorithms excluding IR and MR.

Table 8 presents the variation in $\text {Ag}$ and $\text {Te}$ according to *Ct* for all algorithms, where the average running time is the same for a fixed algorithm and for all Ct values. The maximum average gap of 0.63 is obtained by the IR algorithm when $Ct=\{3,8\}$. For all algorithms, when $Ct=3$ and 8, the average gap reaches maximum values.

Table 9 also presents the variation in $\text {Ag}$ and $\text {Te}$ according to *C* for all algorithms. This table shows that the values of $\text {Ag}$ and $\text {Te}$ are very close for a fixed algorithm and for all values of *C*.

## Conclusion

The COVID-19 pandemic is a serious issue affecting millions of people worldwide. The current management aims to reduce the spread of the virus and provide supportive care for the affected people without fundamental therapeutic interventions. Therefore, there is an urgent need to develop targeted therapies. Understanding the differences between pediatric and adult responses to this virus may help direct immune-based therapeutics. In this study, we designed an example of parking in a smart city and developed nine algorithms to address the problem of vehicle assignment seeking a fair distribution of persons in parking. This fair distribution is a very important issue for COVID-19. The developed algorithms are based on several methods, such as the randomized method, iterative method, clustering method, and dispatching rules method. The experimental results showed the performance of these algorithms in terms of the gap time. The best algorithm was found to be C3S, with an average gap of 0.04. The proposed algorithms can be used to develop more enhanced algorithms by applying several metaheuristics. In addition, the developed algorithms can serve as inputs to develop an exact solution for the studied problem.
